# BERTOLOTTI SYNDROME

**DOI:** 10.1590/1413-785220253305e290072

**Published:** 2025-09-22

**Authors:** Eduardo Achar, João Paulo de Souza Sanches Trecco, Robert Meves, Marcos Vaz de Lima

**Affiliations:** 1 Irmandade Santa Casa de Misericordia de Sao Paulo (ISCMSP), Departamento de Ortopedia e Traumatologia, Pavilhao F. Simonsen, Sao Paulo, SP, Brazil.

**Keywords:** Spine, Low Back Pain, Lumbosacral Region, Coluna Vertebral, Dor Lombar, Região Lombossacral

## Abstract

**Introduction:**

Lower back disorders are prevalent and a significant reason for emergency care visits. In 2020, 619 million people experienced low back pain, expected to rise to 843 million by 2050. A common cause is the presence of a lumbosacral transitional vertebra (LSTV). Objectives: This study evaluates the prevalence of Bertolotti Syndrome in patients with low back pain in an emergency setting and assesses interobserver reliability of LSTV classifications.

**Methods:**

A retrospective analysis of 1023 lumbar spine radiographs from patients presenting with low back pain from 2018 to 2020 was conducted. After exclusions, 469 radiographs were analyzed. Two orthopedists classified LSTVs using Tini and Castellvi's systems. Statistical analyses included the Kappa agreement index, two-proportion Z test, confidence interval for the mean, and p-value calculations.

**Results:**

The prevalence of Bertolotti Syndrome was 62.5% for observer A and 61.6% for observer B. Type I LSTV was the most common, with over 70% of cases, followed by type III at over 15%. The most frequent morphology was bilateral involvement of the transverse process, with more than 50% of cases exhibiting IB morphology according to both classifications.

**Conclusion:**

This study found a high incidence of Bertolotti Syndrome (over 60%) in patients with low back pain seeking emergency care, suggesting that LSTV should be more frequently considered in differential diagnoses. Improved recognition of LSTV could lead to better management strategies for low back pain associated with this congenital anomaly. ***Level of Evidence III; Retrospective Cohort Study.***

## RESUMO

**Introdução:**

Distúrbios na região lombar são prevalentes e uma razão significativa para visitas de emergência. Em 2020, 619 milhões de pessoas experimentaram dor lombar, com expectativa de aumento para 843 milhões até 2050. Uma causa comum é a presença de uma vértebra de transição lumbossacral (LSTV).

**Objetivos:**

Este estudo avalia a prevalência da Síndrome de Bertolotti em pacientes com dor lombar em ambiente de emergência e avalia a confiabilidade entre observadores das classificações de LSTV.

**Métodos:**

Uma análise retrospectiva de 1023 radiografias da coluna lombar de pacientes com dor lombar entre 2018 e 2020 foi realizada. Após exclusões, 469 radiografias foram analisadas. Dois ortopedistas classificaram as LSTVs utilizando os sistemas de Tini e Castellvi. As análises estatísticas incluíram o índice de concordância Kappa, o teste Z de duas proporções, o intervalo de confiança para a média e os cálculos de valor de p.

**Resultados:**

A prevalência da Síndrome de Bertolotti foi de 62,5% para o observador A e de 61,6% para o observador B. A LSTV tipo I foi a mais comum, com mais de 70% dos casos, seguida pelo tipo III com mais de 15%. A morfologia mais frequente foi o envolvimento bilateral do processo transverso, com mais de 50% dos casos exibindo morfologia IB de acordo com ambas as classificações.

**Conclusão:**

Este estudo encontrou uma alta incidência da Síndrome de Bertolotti (mais de 60%) em pacientes com dor lombar que buscaram atendimento de emergência, sugerindo que a LSTV deve ser mais frequentemente considerada nos diagnósticos diferenciais. O reconhecimento aprimorado da LSTV pode levar a melhores estratégias de manejo para a dor lombar associada a essa anomalia congênita. ***Nível de Evidência III; Estudo de Coorte Retrospectivo.***

## INTRODUCTION

Disorders of the lumbar region are among the most common injuries in the general population and are one of the main reasons for seeking emergency care. It is estimated that 619 million people experienced low back pain in 2020, approximately 10% of the global population, and that this number is expected to increase by more than 35% over the next thirty years, reaching 843 million by 2050.^1^ Only a small portion of these patients have well-established causes for the pathology, such as vertebral fracture, malignancy, or infection. Another known and widely discussed cause to justify low back pain is the presence of a lumbosacral transitional vertebra.^2,3^ A transitional vertebra is the most common congenital anomaly of the lumbosacral spine,^4–5^ with a prevalence ranging from 7% to 30% in various studies.^6–7^ It is characterized by an abnormal enlargement of the transverse process of the most caudal lumbar vertebra, which may articulate or fuse with the sacrum unilaterally or bilaterally.^8^ It is caused by ligamentous ossification, chronic arthritis, or true sacralization.^9^

Several studies have reported the presence of Bertolotti's Syndrome in the population,^7–10^ however, they lack data correlating the percentage of patients with low back pain who have the condition.

## METHODOLOGY

A retrospective study of 1,023 anteroposterior lumbar spine radiographs was conducted in patients who sought emergency care between 2018 and 2020 due to low back pain without a history of recent trauma, resulting in a final sample of 469 cases. Radiographs were excluded if they showed: significant spinal deformities, fractures of the vertebral bodies or transverse processes, poorly performed radiographs with overlapping structures or axial rotation that prevented proper analysis, postoperative images of instrumented arthrodesis, or bone resections. The patients were between 11 and 96 years old and were exempted from signing the Informed Consent Form, CAEE 46563815.9.0000.5479.

The radiographs were independently evaluated by two orthopedic surgeons, and the cases were classified using a standardized approach based on the radiographic classification systems of Tini and Castellvi.^2–3^ The reviewers assessed the reproducibility of the descriptions and determined interobserver reliability. The variables were analyzed using the Kappa concordance index, two-proportion Z-test, confidence interval for the mean, and p-value. The classifications used to detail the degrees of involvement of the megatransverse process are divided into grades (I to IV – referred to as "class") and their subdivisions (a, b, c – referred to as "morphology").

**Figure 1:**
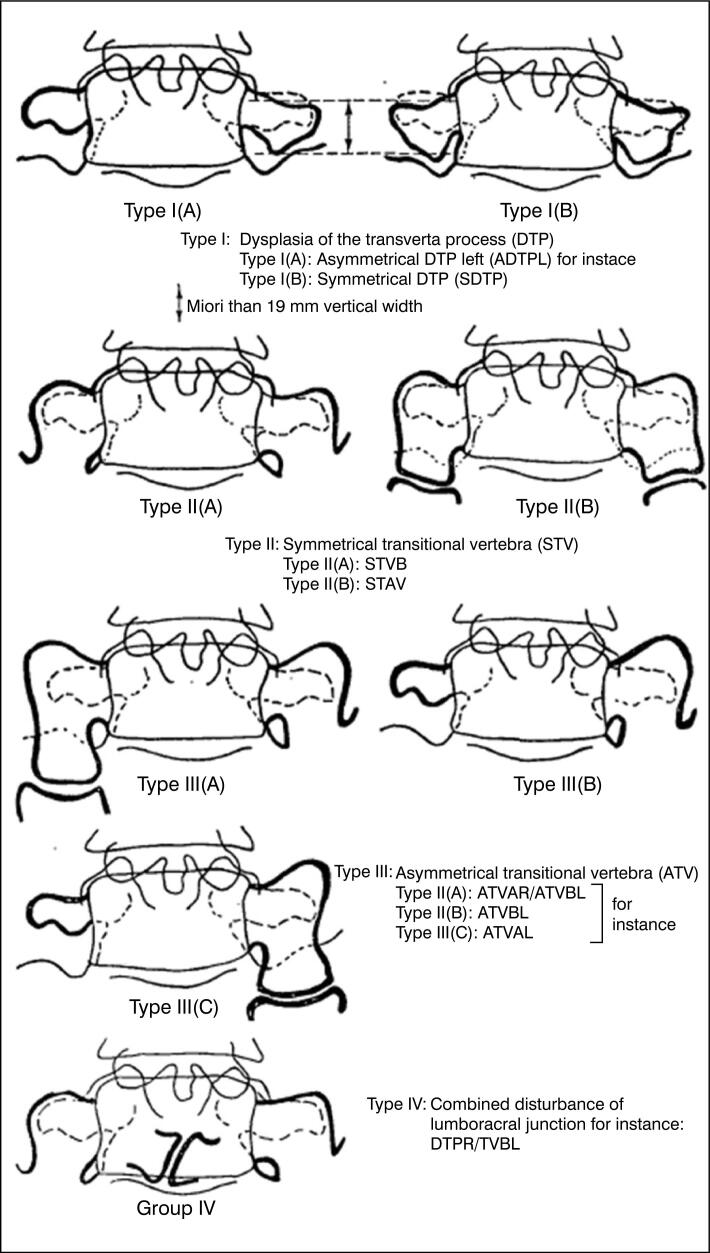
Tini Classification.^2^

In the Tini classification^2^ (Figure 1), type I represents only a change in the length of the transverse process of the presacral vertebra greater than 1.9 cm, which may be asymmetric (a) or symmetric (b) in relation to the contralateral side; type II represents the bilateral and symmetric appearance of the transitional vertebra, which may be fused by a bony bridge (a) or articulated (b) with the sacrum; type III presents the transitional vertebra bilaterally and asymmetrically, which may involve one side articulated and the other fused by a bony bridge with the sacrum (a), one articulated and the other normal (b), or one fused by a bony bridge and the other normal (c); and type IV represents combined disorders, with dysplasia of the transverse process on one side and a new joint or bony bridge on the other side.

**Figure 2:**
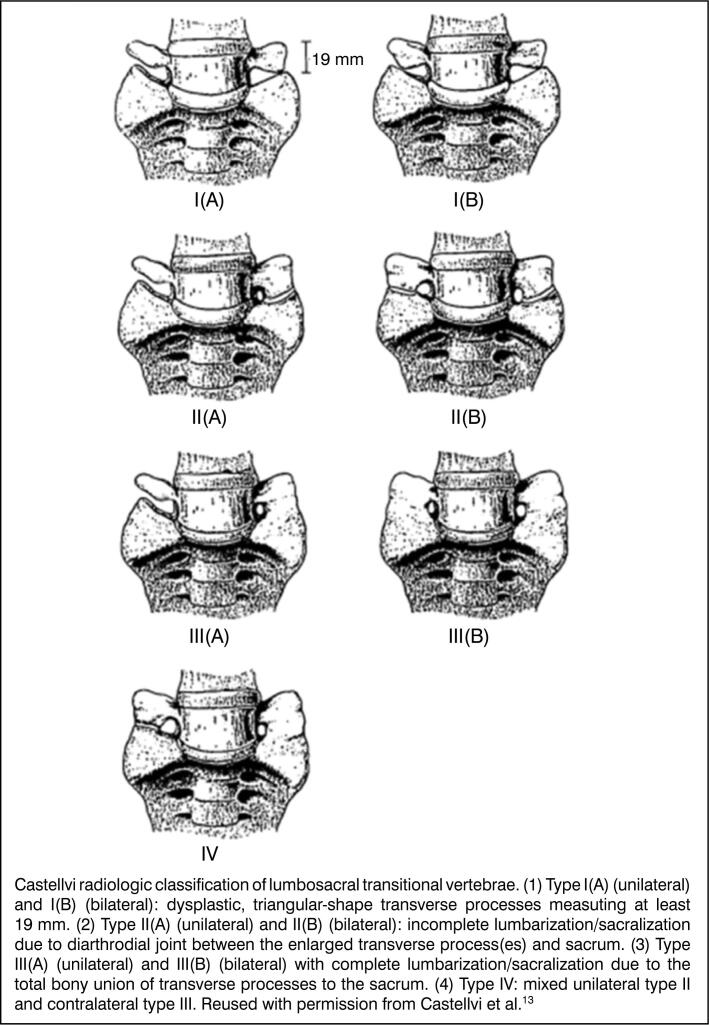
Castellvi Classification.^3^

In the Castellvi classification^3^ (Figure 2), type I represents the presence of hypertrophy of the transverse process measuring at least 1.9 cm, without the formation of a bony bridge or new joint, and may be unilateral (a) or bilateral (b); in type II, a joint is formed between the transverse process and the sacrum, which may be unilateral (a) or bilateral (b); in type III, there is complete sacralization due to bony fusion, which may also be unilateral (a) or bilateral (b); and type IV presents both joint formation and bony fusion simultaneously.

## RESULTS

Based on the data presented and the statistical analyses performed, the main results can be highlighted as follows: data variability was assessed using the coefficient of variation (CV), which was less than 50%, indicating low variability and high homogeneity of the data. The Confidence Interval (CI) was calculated for the mean age (Table 1), with 95% statistical confidence, resulting in a mean of 49.9 years ± 1.6, indicating that the mean age may range from 48.3 to 51.5 years.

**Table 1:** Complete Descriptive Analysis for Age.

Age	
Mean	49.9
Median	48
Standard Deviation	17.9
CV	35.8%
Q1	37
Q3	63
IQR	26
Mode	39
Min	11
Max	96
N	469
CI	1.6

For the analysis of the distribution of qualitative factors (Table 2), a two-proportion Z-test was performed to characterize the distribution of qualitative factors such as sex, syndrome, classes, and morphology, as evaluated by evaluators A and B. The results demonstrated statistical significance in all variables analyzed.

**Table 2:** Distribution of Qualitative Factors.

		N		%		P-value
Sex	Female	289	61.6%		<0.001	
	Male	180	38.4%	
CASTELLVI-A (Syndrome)	No	176	37.5%		<0.001	
	Yes	293	62.5%	
CASTELLV-AI (Classes)	I	217	74.1%		Ref.	
	II	22	7.5%		<0.001	
	III	44	15.0%		<0.001	
	IV	10	3.4%		<0.001	
CASTELLVI-A (Morphology)	IA	55	18.8%		<0.001	
	IB	162	55.3%		Ref.	
	IIA	8	2.7%		<0.001	
	IIB	14	4.8%		<0.001	
	IIIA	26	8.9%		<0.001	
	IIIB	18	6.1%		<0.001	
	IV	10	3.4%		<0.001	
CASTELLVI-B (Syndrome)	No	180	38.4%		<0.001	
	Yes	289	61.6%	
CASTELLVI-B (Classes)	I	213	73.7%		Ref.	
	II	19	6.6%		<0.001	
	III	47	16.3%		<0.001	
	IV	10	3.5%		<0.001	
CASTELLVI-B (Morphology)	IA	50	17.3%		<0.001	
	IB	163	56.4%		Ref.	
	IIA	6	2.1%		<0.001	
	IIB	13	4.5%		<0.001	
	IIIA	25	8.7%		<0.001	
	IIIB	22	7.6%		<0.001	
	IV	10	3.5%		<0.001	
TINI-A (Syndrome)	No	176	37.5%		<0.001	
	Yes	293	62.5%	
TINI-A (Classes)	I	219	74.5%		Ref.	
	II	14	4.8%		<0.001	
	III	44	15.0%		<0.001	
	IV	17	5.8%		<0.001	
TINI-A (Morphology)	IA	57	19.4%		<0.001	
	IB	162	55.1%		Ref.	
	IIA	2	0.7%		<0.001	
	IIB	12	4.1%		<0.001	
	IIIA	17	5.8%		<0.001	
	IIIB	20	6.8%		<0.001	
	IIIC	7	2.4%		<0.001	
	IV	17	5.8%		<0.001	
TINI-B (Syndrome)	No	180	38.4%		<0.001	
	Yes	289	61.6%	
TINI-B (Classes)	I	214	74.0%		Ref.	
	II	13	4.5%		<0.001	
	III	45	15.6%		<0.001	
	IV	17	5.9%		<0.001	
TINI-B (Morphology)	IA	51	17.6%		<0.001	
	IB	163	56.4%		Ref.	
	IIA	1	0.3%		<0.001	
	IIB	12	4.2%		<0.001	
	IIIA	18	6.2%		<0.001	
	IIIB	19	6.6%		<0.001	
	IIIC	8	2.8%		<0.001	
	IV	17	5.9%		<0.001	

Regarding the Kappa concordance index (Table 3), it was calculated to assess the degree of agreement between evaluators A and B for the Castellvi^3^ and Tini^2^ classifications, considering different segmentations by age, sex, and age group. The results showed high levels of agreement, with Kappa values above 0.90 in all analyses performed. In summary, the results indicate significant homogeneity in age data, statistical significance in the distribution of qualitative factors, and high agreement between evaluators for the classifications studied. These findings are important for the understanding and interpretation of results in the healthcare field, highlighting the reliability of the analyses performed.

**Table 3:** Kappa Concordance Index between Evaluators for CASTELLVI^3^ and TINI.^2^

With Syndrome		CASTELLVI			TINI		
		Classes	Morphology	With Syndrome	Classes	Morphology	
Up to 49 years	Kappa	0.966	0.959	0.952	0.957	0.952	0.946
	IC	0.033	0.033	0.032	0.037	0.035	0.034
	P-value	<0.001	<0.001	<0.001	<0.001	<0.001	<0.001
50 or more	Kappa	0.980	0.927	0.911	0.980	0.954	0.929
	IC	0.027	0.041	0.043	0.027	0.034	0.039
	P-value	<0.001	<0.001	<0.001	<0.001	<0.001	<0.001
Fem	Kappa	0.970	0.925	0.919	0.970	0.946	0.928
	IC	0.029	0.037	0.037	0.029	0.032	0.035
	P-value	<0.001	<0.001	<0.001	<0.001	<0.001	<0.001
Masc	Kappa	0.977	0.973	0.953	0.965	0.964	0.953
	CI	0.032	0.031	0.037	0.039	0.035	0.037
	P-value	<0.001	<0.001	<0.001	<0.001	<0.001	<0.001
All	Kappa	0.973	0.943	0.932	0.968	0.953	0.938
	CI	0.022	0.026	0.027	0.023	0.024	0.026
	P-value	<0.001	<0.001	<0.001	<0.001	<0.001	<0.001

Given the analysis of the results, the prevalence of Bertolotti's syndrome for observer A was 62.5%, while for observer B it was 61.6%. Among patients with the study syndrome, the class with the highest prevalence in both classifications was type I with more than 70% of cases. The second most prevalent class was type III, with more than 15% of cases. Among the morphologies, the bilateral pattern of involvement of the transverse process was the most frequent. With more than 50% of the cases, IB morphology, Castellvi^3^ and Tini,^2^ was the most prevalent.

## DISCUSSION

The causal relationship between the transitional vertebra and low back pain was first described in 1917 as Bertolotti's Syndrome.^9^ Several factors support this relationship, as affected individuals have a higher likelihood of degeneration in adjacent segments due to the joint hypomobility generated at the level of the fusion, leading to increased load on the disc immediately above it.^10–13^ Cadaveric studies have shown that the iliolumbar ligament is significantly thinner and weaker at the level above the transitional vertebra.^11^ Additionally, narrowing of the intervertebral foramen caused by the widening of the transverse foramen may lead to nerve root compression.^11^

The incidence of Bertolotti's Syndrome is 4% to 6% in the general population.^11^ However, Quinland et al. reported that when isolating the young population (under 30 years old), the incidence rises to 11%.^8^ The diagnosis is based on radiological findings correlated with the clinical presentation. Normally anteroposterior radiography of the lumbosacral spine is sufficient, but in cases of milder hemisacralization, computed tomography is necessary for identification. In addition, the Magnetic Resonance is used to evaluate the root disturbances.^14^

In our study, patients with low back pain who sought care at the Emergency Department (approximately 60%) also had a transitional vertebra and, therefore, Bertolotti's Syndrome. Of these, the bilateral involvement of the transverse process, especially its hypertrophy without articulation with the sacred, was the most prevalent.

Bertolotti's syndrome is rarely considered as a differential diagnosis for patients presenting to emergency care with lower back pain, both due to physicians’ lack of awareness and the limited dissemination and study of the condition in the literature. Various pathologies such as musculoskeletal distensions, degenerative disc disease, degenerative or rheumatic spinal disorders and spondylolisthesis are possibilities considered before Bertolotti's syndrome.

This study reveals an incidence of over 60% of Bertolotti's Syndrome among the 469 patients with low back pain who sought medical care, leading us to the conclusion that the condition should be more thoroughly considered when treating this population. It is worth noting that the presence of a transitional vertebra should receive greater attention, as it is associated with low back pain and is not always detected. This is particularly important considering that the incidence of this anomaly in the general population is much lower than the incidence of low back pain.

This opens up an even broader range of possibilities to be explored: regarding new approaches to the treatment of the megatransverse process associated with low back pain.
